# Neuronal SLC39A8 deficiency impairs cerebellar development by altering manganese homeostasis

**DOI:** 10.1172/jci.insight.168440

**Published:** 2024-10-22

**Authors:** Eun-Kyung Choi, Luisa Aring, Yujie Peng, Adele B. Correia, Andrew P. Lieberman, Shigeki Iwase, Young Ah Seo

**Affiliations:** 1 Department of Nutritional Sciences, University of Michigan School of Public Health, Ann Arbor, Michigan, USA.; 2 Department of Pathology and; 3 Department of Human Genetics, University of Michigan Medical School, Ann Arbor, Michigan, USA.

**Keywords:** Genetics, Neuroscience, Genetic diseases, Mouse models, Neurodevelopment

## Abstract

Solute carrier family 39, member 8 (SLC39A8), is a transmembrane transporter that mediates the cellular uptake of zinc, iron, and manganese (Mn). Human genetic studies document the involvement of SLC39A8 in Mn homeostasis, brain development, and function. However, the role and pathophysiological mechanisms of SLC39A8 in the central nervous system remain elusive. We generated *Slc39a8* neuron-specific knockout (*Slc39a8*-NSKO) mice to study SLC39A8 function in neurons. The *Slc39a8*-NSKO mice displayed markedly decreased Mn levels in the whole brain and brain regions, especially the cerebellum. Radiotracer studies using ^54^Mn revealed that *Slc39a8*-NSKO mice had impaired brain uptake of Mn. *Slc39a8*-NSKO cerebellums exhibited morphological defects and abnormal dendritic arborization of Purkinje cells. Reduced neurogenesis and increased apoptotic cell death occurred in the cerebellar external granular layer of *Slc39a8*-NSKO mice. Brain Mn deficiency in *Slc39a8*-NSKO mice was associated with motor dysfunction. Unbiased RNA-Seq analysis revealed downregulation of key pathways relevant to neurodevelopment and synaptic plasticity, including cAMP signaling pathway genes. We further demonstrated that Slc39a8 was required for the optimal transcriptional response to the cAMP-mediated signaling pathway. In summary, our study highlighted the essential roles of SLC39A8 in brain Mn uptake and cerebellum development and functions.

## Abstract 2

Our studies establish the essential roles of SLC39A8 in brain manganese homeostasis and cerebellum development and functions.

## Introduction

Acquired through the diet, manganese (Mn) is an essential nutrient required for normal growth and physiological processes, including brain development and function (1). The essential roles of Mn in these processes include its participation as a cofactor for various enzymatic processes, such as the conversion of glutamate to glutamine in the brain via glutamine synthetase (2) and protection against free radicals via Mn superoxide dismutase (3). Brain Mn levels increase after birth, and young rats show the greatest Mn incorporation into the brain (4), implying a vital role of this nutrient in neurodevelopment. Given the narrow range between essential and toxic doses of Mn, both low and high levels of Mn have been associated with adverse effects on children’s neurodevelopment (5–7). Mn deficiency can be a risk factor for epilepsy in both humans and rats (8), whereas Mn toxicity has been reported in occupational settings where miners, welders, and dry manufacturers are exposed to chronic inhalation of high concentrations of respirable airborne Mn (9, 10). Exposure to high levels of Mn can result in Mn accumulation in the brain and result in a parkinsonian-like disorder known as manganism (11).

Recent genetic studies have revealed essential roles for solute carrier family 39, member 8 (SLC39A8), in Mn homeostasis and brain development and function. SLC39A8, also known as ZIP8, is a transmembrane transporter that can mediate the cellular uptake of zinc (12), iron (13), and Mn (14, 15). *SLC39A8* loss-of-function mutations have been identified in patients with inappropriately low blood levels of Mn, but their levels of other metals, including zinc, iron, and cadmium, were normal (16–18). Notably, these patients displayed severe neurological symptoms characterized by intellectual disabilities, developmental delay, cerebellar atrophy, growth abnormalities, and seizures (16–18).

An in vitro study by this research group has shown that SLC39A8 is a cell surface transporter that stimulates ^54^Mn incorporation into cells. The disease-associated mutations completely abrogate the cellular uptake of ^54^Mn (19), thereby providing a causal link between *SLC39A8* deficiency and Mn deficiency. A study of hepatocyte-specific *Slc39a8*-knockout (KO) mice showed that SLC39A8 localizes to the apical membrane of hepatocytes, where it likely functions to reclaim Mn from the bile, suggesting a role for this transporter in hepatic Mn homeostasis (20). In addition to disruptive *SLC39A8* mutations, a common genetic variant in *SLC39A8* (p.Ala391Thr) is associated with multiple phenotypic traits and disease risks, including reduced blood Mn (21), neurological outcomes (including childhood neurodevelopment and behavioral problems) (22), lower fgeneral cognitive function (23), greater risks for schizophrenia (24–26), greater gray matter volume in multiple brain regions (27), and notably, a protective effect against Parkinson’s disease (26). Interestingly, the lead variant has an 8% minor allele frequency in people of European ancestry (28) and has been reported to encode a protein with reduced function (29). Despite this clinical relevance, the lack of knowledge regarding the role and pathophysiological mechanisms of SLC39A8 in the CNS has hindered therapeutic progress.

Brain function and disorders associated with mutations in genes encoding metal ion transporters can be readily assessed in mice because of the evolutionary conservation of the metal homeostasis machinery and the genetic nature of the associated diseases. For example, loss-of-function mutations in *SLC30A10* have been reported in patients with elevated blood Mn levels, Mn accumulation in the liver and brain, and parkinsonism (30–32). *Slc30a10*-deficient mice recapitulate these human phenotypes, including the Mn accumulation in the brain (33, 34). Similarly, loss-of-function mutations in *SLC39A14* have been reported in patients with high blood levels of Mn and accumulation of Mn in the brain (but not in the liver) and with juvenile-onset dystonia-parkinsonism (35), and *Slc39a14*-deficient mice again model these human phenotypes (36, 37). Both SLC30A10 and SLC39A14 are required for Mn homeostasis and, therefore, provide models for understanding the consequences of Mn accumulation in the brain. However, no animal model has yet been reported for exploring Mn deficiency in the CNS, thereby hindering the understanding of Mn regulation in the brain and the development of potential therapies. We have generated *Slc39a8* neuron-specific knockout (*Slc39a8*-NSKO) mice to test the hypothesis that SLC39A8 may affect brain development and function by altering Mn homeostasis, to determine what role *SLC39A8* deficiency plays in the brain and to explore the underlying cellular and molecular mechanisms.

## Results

### Neuronal Slc39a8 deletion leads to reduced Mn levels in the brain.

**Figure 1:**
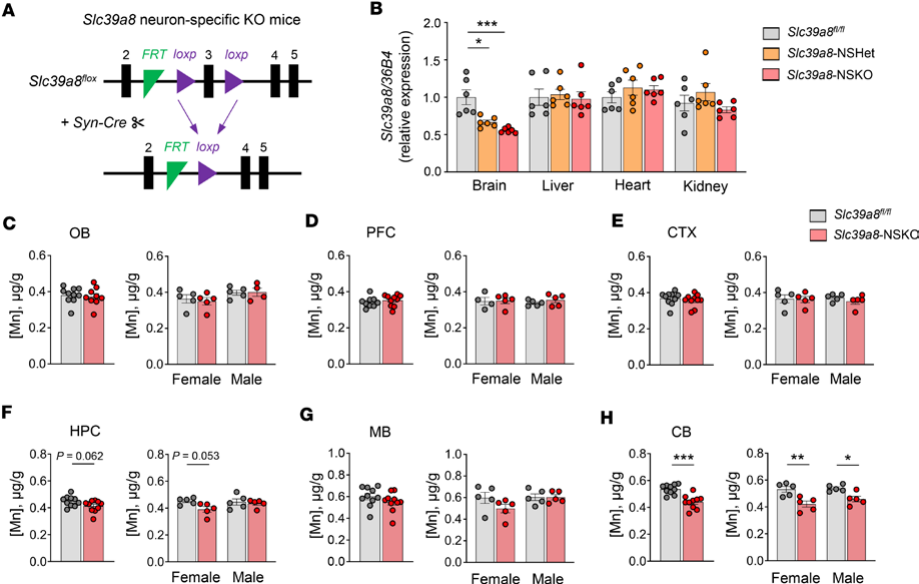
Loss of *Slc39a8* in neurons results in Mn deficiency in the brain region. (**A**) Schematic representation of mice with deletion of *Slc39a8* in the neurons. (**B**) qPCR analysis of *Slc39a8* expression in 4-week-old control and *Slc39a8-*NSKO mice. (**C**−**H**) ICP-MS analysis of Mn levels in olfactory bulbs (OB) (**C**), prefrontal cortex (PFC) (**D**), cortex (CTX) (**E**), hippocampus (HPC) (**F**), midbrain (MB) (**G**), and cerebellum (CB) (**H**) of 4-week-old male and female control and *Slc39a8-*NSKO mice. Data are presented as individual values and represent the mean ± SEM. * *P* < 0.05, ** *P* < 0.01, and *** *P* < 0.001.

To identify the role of SLC39A8 in neurons, we generated *Slc39a8-*NSKO mice by crossing *Slc39a8-flox* (*Slc39a8^fl/fl^*) mice with *Synapsin I-Cre* mice (Figure 1A). The quantitative PCR (qPCR) analysis verified lower *Slc39a8* mRNA levels in the brains of *Slc39a8-*NSKO and *Slc39a8* neuron-specific heterozygous (NSHet) mice (–44.5%, *P* < 0.001 and –33.3%, *P* < 0.05, respectively) than in control *Slc39a8^fl/fl^* mice but normal *Slc39a8* expression in other tissues, including liver, heart, and kidney (Figure 1B). The *Slc39a8-*NSKO mice were born at expected Mendelian ratios and exhibited grossly normal appearance and body weight (Supplemental Figure 1A; supplemental material available online with this article; https://doi.org/10.1172/jci.insight.168440DS1), indicating that neuron-specific deletion of *Slc39a8* did not lead to lethality. We also compared the brain weights of *Slc39a8-*NSKO mice with those of the control mice and observed no substantial differences (Supplemental Figure 1B).

To examine whether the loss of *Slc39a8* in neurons alters metal homeostasis, we used inductively coupled plasma mass spectroscopy (ICP-MS) to measure the concentrations of metals in the whole brain and brain regions from *Slc39a8-*NSKO and control mice. The Mn concentrations were lower in the whole brain of *Slc39a8-*NSKO mice than in the controls (–23.1%, *P* < 0.001) (Supplemental Figure 2A). To further determine whether the regulation of Mn by neuron-specific *Slc39a8* was sex specific, we measured Mn levels in the same tissues of male and female mice. We observed reduced Mn levels in both female (–21.6%, *P* < 0.05) and male (–24.5, *P* < 0.01) *Slc39a8-*NSKO whole brains (Supplemental Figure 2A). We also found that although SLC39A8 can mediate the cellular uptake of zinc (12) and iron (13), the concentrations of these metals did not show any differences (Supplemental Figure 2, B and C). Furthermore, concentrations of Mn and other metals in the liver and whole blood did not differ between the 2 groups (Supplemental Figure 2, D–I).

We next determined whether *Slc39a8*-NSKO brains show region-specific reductions in Mn levels by quantifying the distribution of metals in different brain regions. The Mn concentrations were specifically reduced in the cerebellum (–17.9%, *P* < 0.001) of both female (–15.1%, *P* < 0.01) and male (–19.3%, *P* < 0.05) *Slc39a8*-NSKO mice (Figure 1, C–H). The zinc concentration in the female *Slc39a8*-NSKO hippocampus was reduced (–8.7%, *P* < 0.05; Supplemental Figure 3); however, the overall concentrations of zinc and iron in the brain regions did not differ between the 2 groups (Supplemental Figure 3). Taken together, our *Slc39a8*-NSKO mouse model results indicate that neuron-specific deletion of *Slc39a8* leads to brain Mn deficiency, especially in the cerebellum, with little impact on other metal levels.

### Slc39a8-NSKO mice display impaired brain uptake of ^54^Mn.

**Figure 2:**
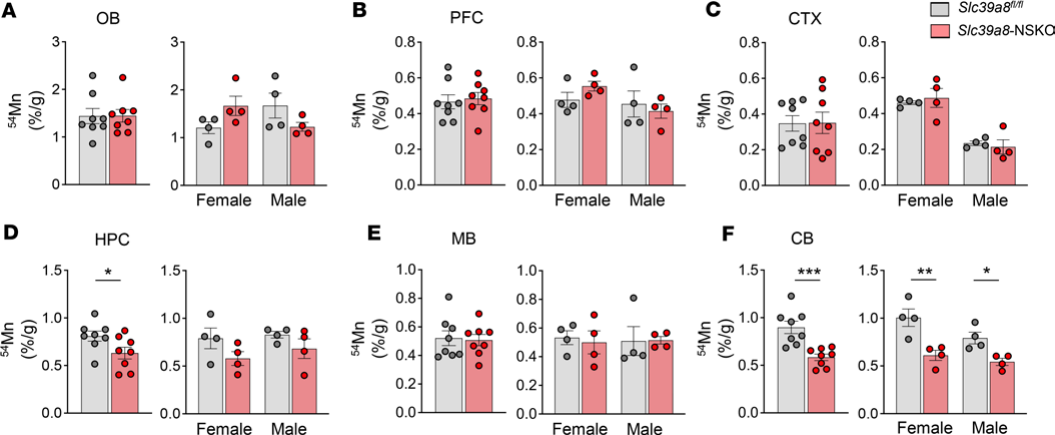
Loss of *Slc39a8* in neurons results in impaired brain Mn uptake. Control and *Slc39a8-*NSKO mice at 4 weeks of age were administered 0.1 μCi [^54^Mn]MnCl_2_ per gram body weight via tail vein injection. Brain regions were collected at 1 hour, and ^54^Mn uptake was determined with a gamma counter (counts per minute [cpm]). Levels of ^54^Mn in olfactory bulbs (OB) (**A**), prefrontal cortex (PFC) (**B**), cortex (CTX) (**C**), hippocampus (HPC) (**D**), midbrain (MB) (**E**), and cerebellum (CB) (**F**) from 4-week-old male and female control and *Slc39a8-*NSKO mice. Data are presented as individual values and represent the mean ± SEM. * *P* < 0.05, ** *P* < 0.01, and *** *P* < 0.001.

The lower Mn concentrations in the whole brain and brain regions of the *Slc39a8-*NSKO mice (Figure 1 and Supplemental Figure 2) suggested that *Slc39a8*-NSKO mice might have a defect in brain Mn uptake. We tested this possibility by measuring radioactivity in the brain regions of control and *Slc39a8*-NSKO mice at 1 hour after intravenous injection of ^54^Mn. The 1-hour time point was chosen based on previous studies of brain uptake of Mn in mice (38, 39). We observed that brain ^54^Mn levels were substantially reduced in the hippocampus (–22%, *P* < 0.05) and cerebellum (–35%, *P* < 0.001) in the *Slc39a8-*NSKO mice compared with control mice (Figure 2). Notably, the amount of ^54^Mn in the cerebellum was reduced in both female (–21.2%, *P* < 0.01) and male (–11.1%, *P* < 0.05) *Slc39a8-*NSKO mice compared with control mice (Figure 2).

Animals acquire Mn primarily through diet under normal conditions. We also measured radioactivity in the brain regions of control and *Slc39a8*-NSKO mice 1 hour after intragastric gavage of ^54^Mn. The amount of ^54^Mn in the cerebellum was reduced by 35.4% (*P* < 0.01) in *Slc39a8-*NSKO mice compared with control mice (Supplemental Figure 4, A–F). The amount of ^54^Mn in the blood was similar between control and *Slc39a8-*NSKO mice 1 hour after administration of ^54^Mn via intravenous injection or intragastric gavage (Supplemental Figure 4, G and H). The total amount of ^54^Mn was also measured similarly between control and *Slc39a8*-NSKO mice, verifying uniform exposure conditions (Supplemental Figure 4, I and J). The residual ^54^Mn levels observed in the *Slc39a8*-NSKO cerebellum suggest an alternative entry route for Mn into neurons. To address this, we examined the expression of other metal ion transporters that might compensate for Mn uptake. Notably, the cerebellum of *Slc39a8*-NSKO male mice exhibited an upregulation in *Slc39a14*/ZIP14 transcript levels, although this difference did not reach statistical significance (Supplemental Figure 5, *P* = 0.0649).

No substantial changes were observed in other tested metal ion transporters, including *Slc30a10/ZnT10*, *Slc11a2/DMT1* (divalent metal transporter 1), and *Slc40a1/FPN* (ferroportin-1) transcript levels (Supplemental Figure 5). These findings suggest that Slc39a14 may compensate for the loss of SLC39A8 expression. Overall, these data indicate that neuronal *Slc39a8* deficiency impairs brain ^54^Mn uptake.

### Cerebellar morphological defects in Slc39a8-NSKO mice.

**Figure 3:**
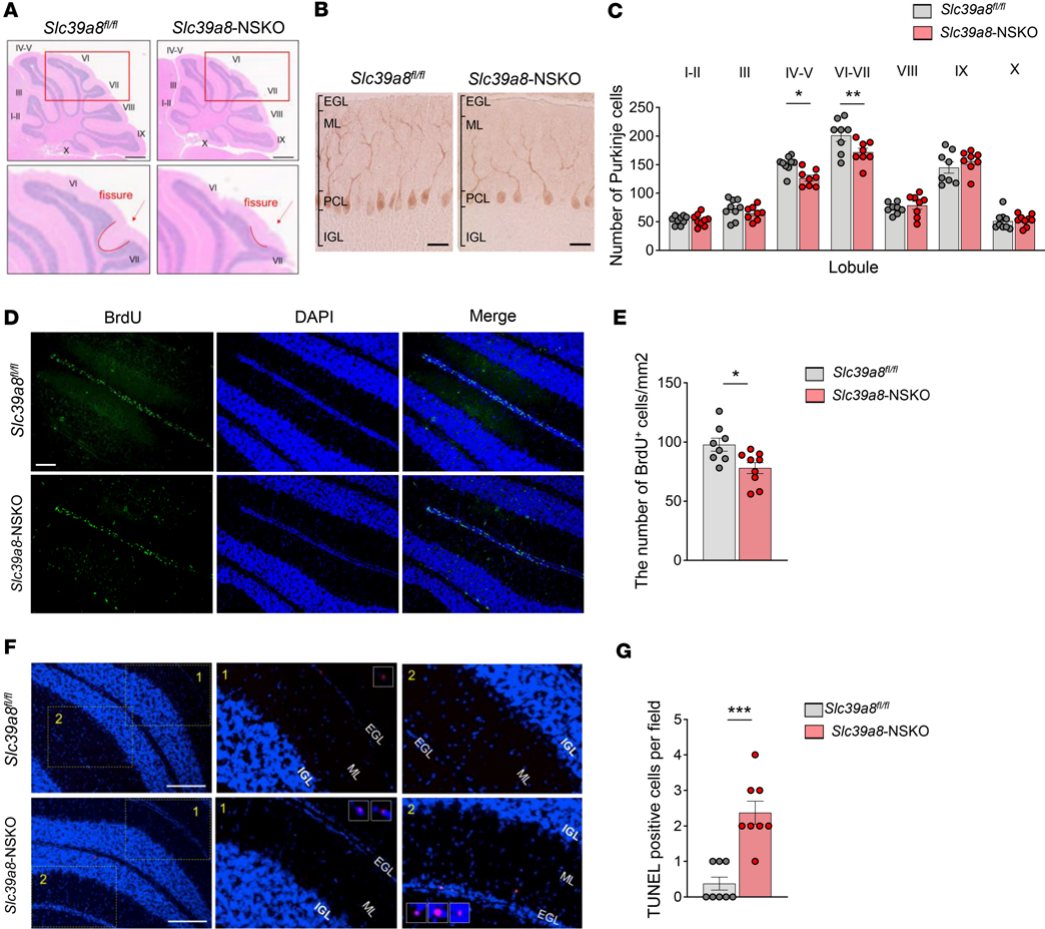
Morphological defects, reduced neurogenesis, and accelerated apoptosis in *Slc39a8*-NSKO cerebellum. (**A**) H&E-stained sagittal sections of paraffin-embedded mouse brains from 4-week-old control and *Slc39a8-*NSKO mice. Numerals indicate the lobules, and the fissures between lobules VI and VII of cerebellums are outlined with red lines and indicated by red arrows. Scale bars: 1,000 μm. Upper original magnification, 2×, and lower original magnification, 10×. (**B**) Immunohistochemical staining of cerebellar Purkinje cells (PCs) with anti-calbindin antibody. PC morphologies of control and *Slc39a8*-NSKO mice at P14. EGL, external granule cell layer; ML, molecular layer; PCL, Purkinje cell layer; IGL, internal granule cell layer. Scale bars: 25 μm. (**C**) Numbers of PCs at P14. (**D**) BrdU staining on the cerebellar sections at P14. Scale bars: 100 μm. (**E**) The number of BrdU^+^ cells per unit size of sagittal sections from the entire cerebellum was quantified. (**F**) TUNEL staining on the cerebellar sections at P14. Scale bars: 200 μm. Insets original magnification, 20×. (**G**) Quantification of the number of TUNEL-positive cells per field of cerebellar folia. At least 9 sections from 3 animals per genotype were quantified for all panels. Data are presented as individual values and represent the mean ± SEM. * *P* < 0.05, ** *P* < 0.01, and *** *P* < 0.001.

We next sought to determine whether the loss of *Slc39a8* in neurons leads to alterations in overall brain architecture. The appearance of the brain of *Slc39a8*-NSKO mice revealed no gross abnormalities. However, systematic histological analysis revealed a striking difference in the foliation and fissuration of the cerebellum in *Slc39a8*-NSKO mice at P28 (Figure 3A). In control mice, the intercrural fissure normally separates lobules VI and VII, but this fissure was absent in the *Slc39a8*-NSKO cerebellum (Figure 3A, indicated by red arrows and red outlines). The observed cerebellar morphological defects were indeed present in multiple *Slc39a8*-NSKO mice at P28, albeit with incomplete penetrance (Supplemental Figure 6). These results suggest a critical role for *Slc39a8* in cerebellar development.

We also examined the morphological abnormalities in the cerebellum of *Slc39a8*-NSKO mice by staining the Purkinje cells (PCs) with an anti–calbindin D antibody, a specific marker for this cell type, at P8 (Supplemental Figure 7A) and P14 (Figure 3B). The cerebellar cortex in the control and *Slc39a8*-NSKO mice displayed the same orderly, 4-layered structural organization of the EGL, ML, PCL, and IGL (40) (Supplemental Figure 7A and Figure 3B). However, the numbers of PCs were noticeably reduced in *Slc39a8*-NSKO mice at both P8 and P14, with lobules IV and VII exhibiting the most significant reductions (Supplemental Figure 7B and Figure 3C). These results indicate that abnormal cerebellar histogenesis occurs during early postnatal development in *Slc39a8*-NSKO mice.

### Reduced neurogenesis and accelerated apoptosis in the EGL in Slc39a8-NSKO cerebellum.

The mouse cerebrum reaches near maturity at birth, whereas the cerebellum continues to grow postnatally (41, 42). During postnatal development, the granule cell precursors (GCPs) proliferate, differentiate into mature granule cells, and migrate to form the IGL (41). Recognizing that cerebellar morphogenesis largely depends on GCP proliferation (41), we sought to determine whether the morphological defects in the *Slc39a8*-NSKO cerebellum can be explained mechanistically through decreased cell proliferation or increased apoptosis. We performed a BrdU staining to examine whether GCPs are altered in the developing cerebellum of *Slc39a8*-NSKO mice at P14. We observed a reduction in the number of BrdU-positive cells in *Slc39a8*-NSKO mice compared with controls (Figure 3, D and E). We then performed a terminal deoxynucleotidyl transferase–mediated dUTP nick end labeling (TUNEL) assay to examine whether neuronal cell death is altered in the developing cerebellum of *Slc39a8*-NSKO mice at P14. The number of TUNEL-positive cells was higher in the EGL of *Slc39a8*-NSKO mice than in the controls (Figure 3, F and G). These results indicate that deletion of *Slc39a8* in neurons impairs neurogenesis and accelerates apoptotic cell death in the EGL of the cerebellum, suggesting an association with the defects in cerebellar morphogenesis of *Slc39a8*-NSKO cerebellum.

### Loss of Slc39a8 in neurons results in defects in dendritic arborization and spine morphology in PCs.

**Figure 4:**
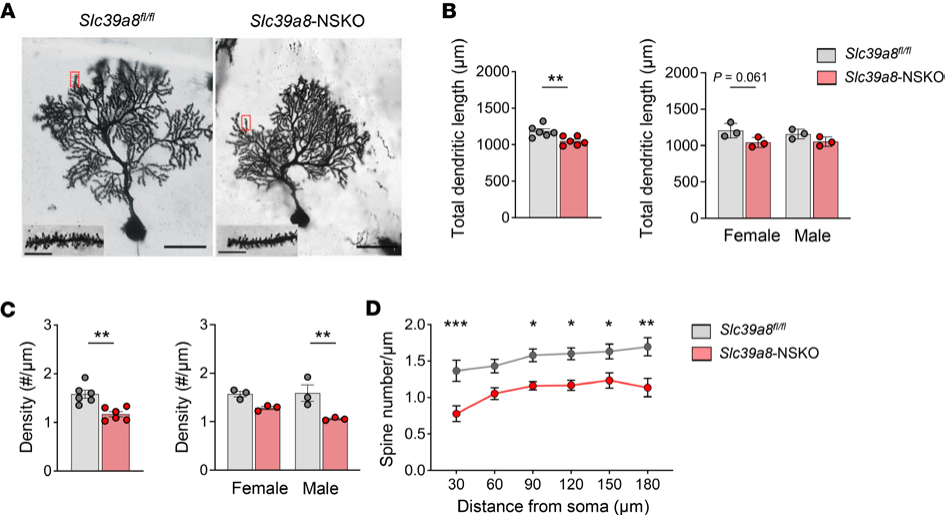
Impaired dendritic arborization of PCs in *Slc39a8*-NSKO cerebellum. (**A**) Representative images of Golgi-stained PCs from control and *Slc39a8*-NSKO mice cerebellum at P28. Scale bars: 50 μm (left) and 5 μm (right). (**B**−**D**) Quantification of total dendritic lengths (**B**), density (**C**), and spine number (**D**) in male and female control and *Slc39a8-*NSKO mice. At least 6 sections from 3 animals per genotype were quantified for all panels. Data are presented as individual values and represent the mean ± SEM. * *P* < 0.05, ** *P* < 0.01.

Abnormal dendritic arborization and spine morphology have been suggested as a cellular basis of neurodevelopmental disorders (43). The role of *Slc39a8* in dendritic growth and spine morphogenesis is not known. Thus, we sought to determine whether dendritic growth in the cerebellum is affected by *Slc39a8* deficiency. Cerebellum impregnated by Golgi staining clearly exhibited a severe reduction in the extent of the spiny dendritic arborization of individual *Slc39a8*-NSKO PCs compared with controls (Figure 4A). Many dendritic spines on the *Slc39a8*-NSKO neurons were remarkably thinner than those of control neurons and lacked the mature mushroom-like morphology (Figure 4A). Golgi staining followed by Sholl analysis showed a shorter total dendritic length in *Slc39a8*-NSKO mice than in the controls (–11.3%, *P* < 0.01, Figure 4B). The *Slc39a8*-NSKO mice also exhibited lower spine density (–25.9%, *P* < 0.01, Figure 4C). Moreover, the reduced spine density in the *Slc39a8*-NSKO mice was evident from the proximal to distal segments of dendritic branches (Figure 4D). These results suggest that developmental defects in the PCs of *Slc39a8*-NSKO mice affect the fine structure of dendritic arborization.

### Slc39a8-NSKO mice exhibit impaired motor coordination.

**Figure 5:**
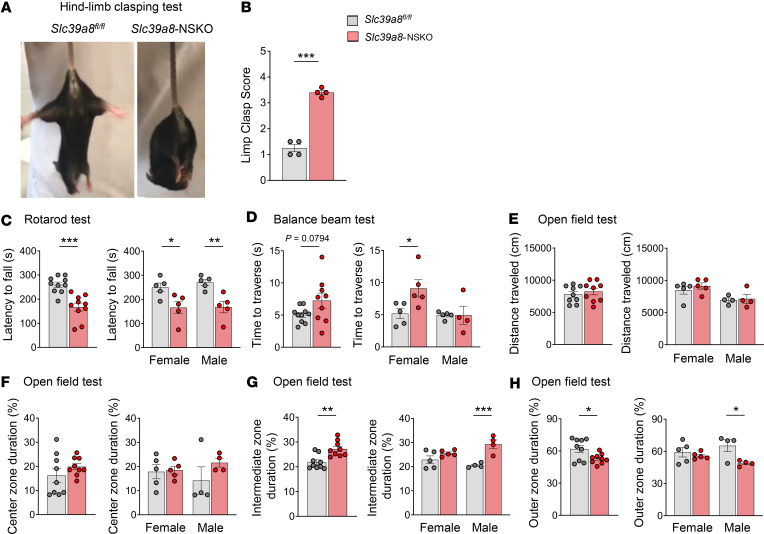
Motor dysfunction in *Slc39a8*-NSKO mice. (**A**) Representative images of a 4-week-old control and *Slc39a8*-NSKO mouse during tail suspension in the clasping test. (**B**) Quantitative analysis of the hind limb clasping scores. (**C**) Rotarod performance of 4-week-old male and female control and *Slc39a8*-NSKO mice. (**D**) Balance performance of 4-week-old male and female control and *Slc39a8*-NSKO mice. (**E**–**H**) Open field test of 4-week-old male and female control and *Slc39a8*-NSKO mice. Distance traveled (**E**), center zone duration (%) (**F**), intermediate zone duration (%) (**G**), and outer zone duration (%) (**H**). Data are presented as individual values and represent the mean ± SEM. * *P* < 0.05, ** *P* < 0.01, and *** *P* < 0.001.

The morphological defects observed in the *Slc39a8*-NSKO cerebellum prompted us to investigate the impact on cerebellar motor function. To assess motor function comprehensively, we applied 4 behavioral tests: the hind limb clasping test, the rotarod test, the balance beam test, and the open field test. The hind limb clasping test measures the occurrence of limb clasping during tail suspension, serving as a functional test for corticospinal deficits (44). Reduced hind limb reflexes were observed in *Slc39a8*-NSKO mice compared with controls (Figure 5, A and B, *P* < 0.001). This indicates a potential deficit in corticospinal function. Next, we evaluated motor coordination using the rotarod test. In this test, mice were placed on a rotating rod with gradually accelerating speed, and the time they stayed on the rod before falling off (retention time) was used as an indicator of overall motor coordination (45). *Slc39a8*-NSKO mice exhibited a shorter latency on the rod compared with controls (Figure 5C, *P* < 0.001), suggesting impaired motor coordination. The balance beam test assesses fine motor skills and balance by requiring mice to walk across an elevated narrow beam to reach a safe platform (46). The overall performance on the balance beam was reduced in *Slc39a8*-NSKO mice, though this difference did not reach statistical significance (Figure 5D, *P* = 0.0794). However, when the data were analyzed by sex, the female *Slc39a8*-NSKO mice showed a significant reduction in the ability to traverse the balance beam (Figure 5D, *P* < 0.05). This highlights a sex-dependent effect on fine motor skills and balance. The open-field assay involved placing the mice in a chamber equipped with sensors to monitor their movement for 30 minutes (47). The overall distance traveled and center zone duration (%) were similar between the genotypes across sexes (Figure 5, E and F). Notably, *Slc39a8*-NSKO mice exhibited a longer duration (%) in the intermediate zone (Figure 5G, *P* < 0.01) and the outer zone (Figure 5H, *P* < 0.05), with a more pronounced effect in male *Slc39a8*-NSKO mice (Figure 5, G and H). These results suggest altered exploratory behavior and anxiety-like behavior, particularly in males. In summary, our integrated analysis of the neurobehavioral tests reveals that *Slc39a8*-NSKO mice exhibit a range of motor function deficits, including impaired corticospinal function, motor coordination, and fine motor skills. Additionally, there are sex-dependent sensitivities, with female mice showing significant impairments in balance and male mice exhibiting altered anxiety-like behavior. These findings collectively underscore the broad and sex-biased impact of *Slc39a8* deficiency on motor function and behavior.

### Slc39a8-NSKO cerebellum shows altered transcriptome profiles.

**Figure 6:**
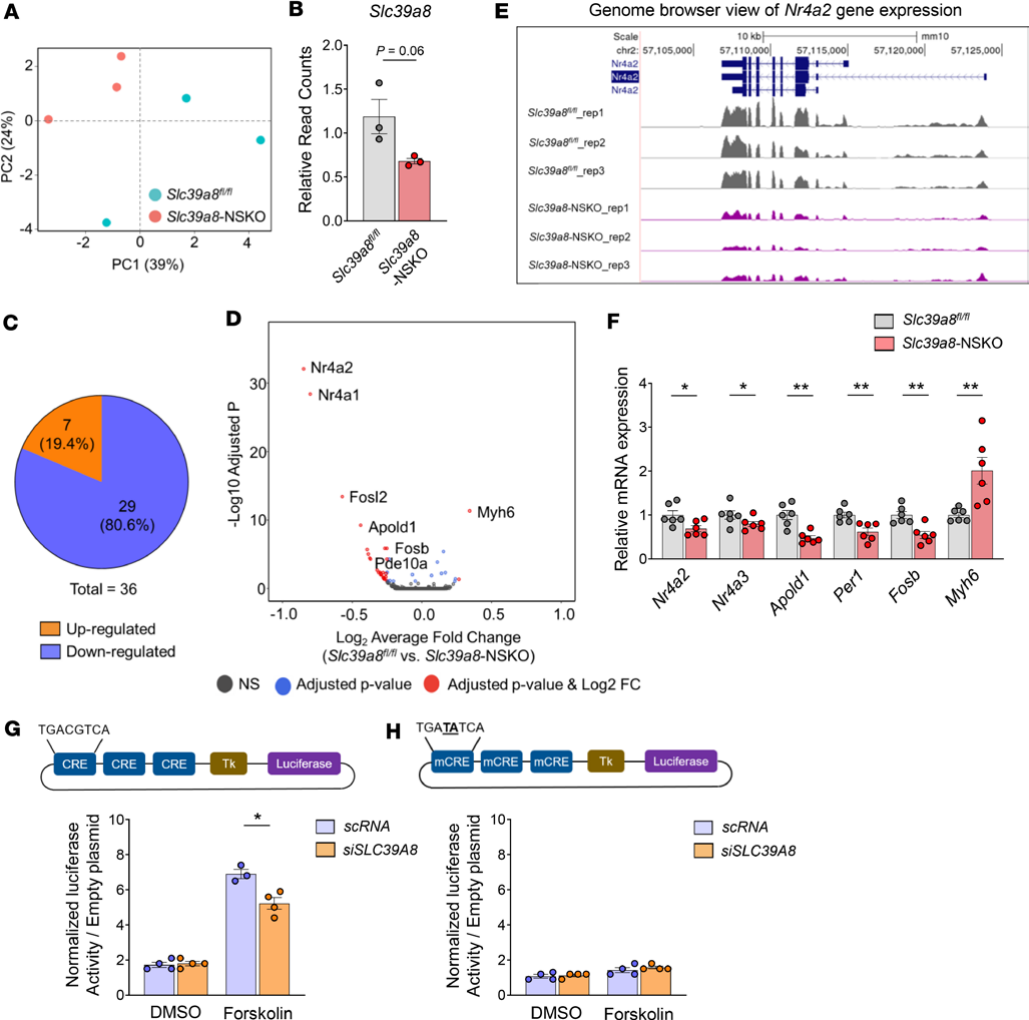
Altered transcription profiles in 4-week-old male *Slc39a8*-NSKO cerebellum. (**A**) Principal component analysis (PCA) plot showing the clustering of each of the samples with technical triplicates along 2 principal components (PC1 — 39% variance; PC2 — 24% variance). Each technical triplicate clusters with the other. (**B**) Relative read counts of *Slc39a8*. (**C**) A total of 29 genes (80.6%) are downregulated and 7 genes (19.4%) are upregulated. (**D**) Volcano plot profiles of –log_10_ *P*adj value and log_2_ fold-change of gene expression between control and *Slc39a8*-NSKO cerebellum samples. (**E**) UCSC Genome Browser shot of the *Nr4a2* locus. (**F**) qPCR validation of *Nr4a2*, *Nr4a3*, *Apold1*, *Per1*, *Fosb*, and *Myh6*, 6 genes that were shown to be dysregulated in the RNA-Seq analysis of the cerebellum of control and *Slc39a8*-NSKO mice. (**G**) A luciferase construct with 3 cAMP-responsive elements (CRE: TGACGTCA) inserted upstream of the HSV–thymidine kinase (HSV-TK) promoter. SH-SY5Y cells were cotransfected with the luciferase construct and expression plasmids for SLC39A8 or scramble siRNAs. cAMP signaling was elicited by the treatment of forskolin, a cAMP analog, and the luciferase activity was measured. (**H**) The mutated 2 critical nucleotides in the CRE sequence (mCRE: TGATATCA) were used as a negative control. The same experiment was performed using the mutated (mCRE) reporter. Data are presented as individual values and represent the mean ± SEM (*n* = 3 samples/group). * *P* < 0.05, ** *P* < 0.01.

To interrogate the molecular mechanisms contributing to impaired cerebellar development, we determined the impact of *Slc39a8* neuronal deficiency on the cerebellum transcriptome. We focused on the cerebellum because *Slc39a8*-NSKO cerebellum showed a pronounced reduction in Mn levels (Figure 1), impaired brain Mn uptake (Figure 2), abnormal development (Figure 3), and defects in spine morphogenesis (Figure 4). We used male mice to include Y-linked genes along with all other chromosomes. A principal component analysis (PCA) plot showed that the 3 replicates clustered together and were segregated into 2 genotype groups, indicating that *Slc39a8* neuronal deficiency triggered transcriptomic alterations (Figure 6A). *Slc39a8* was downregulated in the *Slc39a8*-NSKO cerebellum, verifying the validity of the RNA-Seq approach (Figure 6B). Differentially expressed genes in *Slc39a8*-NSKO and control mice were determined by DESeq2 (48). A total of 36 genes were differentially regulated in the cerebellum of *Slc39a8*-NSKO mice, with an adjusted *P* value (*P*adj) < 0.05 (Supplemental Table 1). Of these, 7 genes were upregulated (19.4%), and 29 genes were downregulated (80.6%) (Figure 6, C and D). The downregulated genes with the 15 largest log_2_ fold-changes are presented in Table 1. A UCSC Genome Browser shot of the *Nr4a2* locus shows a decreased read density in the *Slc39a8*-NSKO mice compared with the controls (Figure 6E). Transcripts that were reduced in the *Slc39a8*-NSKO cerebellum included genes related to CNS development, neuronal differentiation, neurite outgrowth, neuronal survival, circadian rhythms, and CNS myelination (Table 1). The transcripts that increased in the *Slc39a8*-NSKO cerebellum included genes involved in muscle contraction, proteolysis, and transcription regulation (Supplemental Table 1). A qPCR analysis using an independent set of RNA samples verified the downregulation of *Nr4a2*, *Nr4a3*, *Apold1*, *Per1*, and *Fosb* and the upregulation of *Myh6* (Figure 6F). These results indicate that Slc39a8 neuronal deficiency leads to the misregulation of specific genes in the cerebellum.

### Slc39a8 is required for optimal transcriptional response to the cAMP-mediated signaling pathway.

Global transcriptome analysis revealed that cAMP-related genes were among the top downregulated genes (Table 1). The cAMP signaling pathways play crucial roles in diverse physiological processes, such as neurodevelopment, synaptic plasticity, and neuroprotection (49). The cAMP signaling elicited by extracellular stimuli leads to phosphorylation of the transcription factor, cAMP response element binding protein (CREB), and the expression of CREB target genes, resulting in multiple physiological functions (50). For example, CREB regulates cell proliferation, differentiation, and survival in the developing brain while it participates in neuronal plasticity, learning, and memory in the adult brain (51). Notably, the *Slc39a8-*NSKO cerebellum showed downregulation of key genes whose products mediate cAMP signaling (Table 1). Examples of downregulated genes included the NR4A family genes *Nr4a1*, *Nr4a2*, and *Nr4a3*; the FOS families *Fos*, *Fosb*, and *Fosl2*; and the circadian clock gene *Per1*, which all have well-characterized roles as CREB-responsive inducible genes (52, 53).

The observation of downregulation of cAMP signaling mediators and CREB target genes in *Slc39a8-*NSKO cerebellum (Table 1) prompted us to test whether *Slc39a8* deficiency alters the induction of cAMP-mediated gene transcription. We used a reporter plasmid with 3 cAMP-responsive elements (CRE: TGACGTCA) upstream of the firefly luciferase gene (54), as well as a reporter plasmid with mutations in 2 critical nucleotides in the CRE sequence (mCRE: TGATATCA) as a negative control (54). These luciferase constructs and *SLC39A8* or scramble siRNA were transfected into human neuroblastoma SH-SY5Y cells, and the treatment with forskolin, a cAMP analog, stimulated cAMP signaling. The luciferase reporter assay revealed no changes in the basal activity of the CRE-luciferase construct by *SLC39A8* knockdown (Figure 6G). However, following forskolin stimulation, cells with *SLC39A8* siRNA could not induce as high an expression of CRE-luciferase as was observed in the scramble siRNA–treated cells (Figure 6H). The construct with the mutated CRE sequence did not respond to forskolin or change upon *SLC39A8* knockdown, indicating that this effect is cAMP/CREB dependent. These results demonstrate that SLC39A8 is required for the transcriptional response to cAMP-mediated signaling.

## Discussion

We created a mouse model of Slc39a8 neuronal deficiency to explore its role in Mn homeostasis and brain development. Our studies revealed the crucial roles of neuronal Slc39a8 in controlling brain Mn homeostasis and guiding cerebellum development and function. Deletion of *Slc39a8* in neurons impairs brain Mn uptake, reducing Mn content in the whole brain, especially in the cerebellum. Radiotracer ^54^Mn studies verified that Slc39a8 is essential for brain Mn uptake. *Slc39a8* deficiency in neurons led to cerebellar developmental issues, including fissure loss, impaired PC dendritic arborization, and cell-level deficits like reduced neurogenesis and increased apoptosis. Moreover, *Slc39a8*-NSKO mice exhibited impaired motor coordination, indicating abnormal cerebellar functioning. Our unbiased RNA-Seq analysis of the cerebellum of *Slc39a8-*NSKO mice revealed substantial dysregulation of cAMP-related genes. We further demonstrated that Slc39a8 is required for the optimal transcriptional response to the cAMP-mediated signaling pathway. Our studies establish that neuronal Slc39a8 contributes to brain Mn homeostasis by controlling brain Mn uptake and that, through this mechanism, SLC39A8 maintains cerebellum development and function.

A key finding of our study is that Slc39a8 in neurons plays an essential role in controlling brain Mn homeostasis. SLC39A8 is a transmembrane transporter that can mediate the cellular uptake of the essential metals zinc, iron, and Mn, as well as the nonessential metal cadmium (12–15, 55). Recent studies have revealed an essential role for SLC39A8 in Mn homeostasis, as human patients with *SLC39A8* loss-of-function mutations have abnormally low whole-blood Mn levels but normal levels of other metals zinc, iron, and cadmium (16–18). Human carriers of *SLC39A8* A391T, associated with reduced SLC39A8 activity, also showed reduced whole-blood Mn levels (56, 57). A study of hepatocyte-specific *Slc39a8*-KO mice showed that SLC39A8 is essential for hepatic and whole-body Mn homeostasis without affecting other metal levels (20). Studies using *Slc39a8* A391T–knockin mice also showed altered Mn and other metal levels, though the tissue metal levels were variable across the reports (5–9). This variability could be attributed to several factors, including differences in chow formulations, genetic backgrounds, experimental conditions, and other relevant variables. Notably, chow formulations and Mn content across these reports are variable (Supplemental Table 2). In the present study, our systematic analysis of Mn, zinc, and iron in the whole brain and specific brain regions (*n* = 10 per group in both sexes) revealed that the loss of *Slc39a8* in neurons reduced the Mn levels in the cerebellum, without affecting the zinc and iron concentrations. Furthermore, our radiotracer studies using ^54^Mn verified that *Slc39a8*-NSKO mice have impaired Mn uptake in different brain regions, especially the cerebellum, indicating that Slc39a8 mediates brain Mn uptake. Our findings in neuron-specific *Slc39a8*-KO mice suggest that Mn is a primary physiological substrate of SLC39A8 and that SLC39A8 is required for neuronal Mn homeostasis.

Another key finding of our study is that neuronal SLC39A8 is required for cerebellar development and function. SLC39A8 is ubiquitously expressed in the adult brain but shows higher expression in the developing brain than in adult brains in mice (58). During the first 3 weeks of the postnatal mouse life, the cerebellum undergoes dramatic developmental changes: GCPs proliferate in the EGL and differentiate into cerebellar granular cells that migrate to the IGL (41). These developmental changes lead to the formation of the mature cerebellar structure, including the fissure and folia. In contrast with granular cells, PCs cease proliferation at birth and extend their dendrites into the ML, forming synapses from P3 to P28 (59, 60). In the present study, *Slc39a8*-NSKO mice exhibited many deficits in cerebellar development, including a loss of the intercrural fissure between lobules VI and VII and impaired dendritic arborization of PCs. Notably, the lobules VI and VII are associated with the control of balance, eye movements, and coordination (61, 62), suggesting that the impaired motor coordination control observed in *Slc39a8*-NSKO mice might originate from this structural abnormality. Moreover, the cerebellar Mn deficiency in the *Slc39a8*-NSKO mice suggests that SLC39A8 may mainly affect cerebellar development through Mn homeostasis.

These findings are consistent with human genetic studies, where *SLC39A8* mutations were associated with low blood Mn levels and severe neurological symptoms, including cerebellar atrophy (16–18). Our study also aligns with recent GWAS that identified genetic links between SLC39A8 and brain structure/function (27). Our observations of cerebellar defects and abnormal dendritic arborization in *Slc39a8*-NSKO mice suggest a potential mechanistic connection to these genetic associations. In addition, a study reported that individuals carrying a common variant of the *SLC39A8* gene exhibited changes in the T2-weighted to T1-weighted brain signal ratio that varied depending on the number of copies of the allele, further supporting the impact of SLC39A8 on brain structure (63). Our findings of structural changes in the cerebellum in *Slc39a8*-NSKO mice corroborate these human observations, underscoring the relevance of our mouse model in understanding SLC39A8-related cerebellar pathology.

The observed protection against Parkinson’s disease (PD) (26) and insights from human MRI data (57) suggest a potential impact of SLC39A8 on the globus pallidum and substantia nigra, which are critical regions associated with PD (64). However, our study was primarily designed to investigate the role of Slc39a8 in neurons, specifically focusing on its consequences in the cerebellum, resulting in motor dysfunction and cerebellar defects. We did not observe substantial changes in Mn levels or neuropathological alterations in the midbrain, as our study primarily centered on the cerebellum, which houses the majority of neurons. Further investigations warrant exploring potential connections between SLC39A8 deficiency, Mn alterations, and PD. Publicly available single-cell transcriptome analysis data from the human brain (65, 66) demonstrate high SLC39A8 expression in brain endothelial cells (Supplemental Figure 8). However, it is important to note that neurons are the predominant cell type in the brain, and our study primarily focused on their role. While we acknowledge the significance of SLC39A8 in endothelial cells, further studies are required to explore the role of SLC39A8 on various brain cell types. We also acknowledge the limitations of our study. First, *Synapsin I-Cre* (*Syn-Cre*) may exhibit variable expression across brain regions (67, 68). This variability could contribute to potential differential anatomical effects within the brain. Second, we only examined a single developmental time point (4 weeks). Our choice of mice at 4 weeks of age was based on the observed cerebellar abnormalities at P28. While this age selection aligns with our study objectives, future investigations could explore potential age-related factors affecting brain Mn homeostasis or functions.

Our unbiased transcriptome analysis in the *Slc39a8*-NSKO cerebellum revealed the downregulation of pathways crucial for brain development and function, including the cAMP signaling pathway. We showed a requirement for SLC39A8 to induce a CRE-Luciferase reporter gene and a delayed transcriptional response to cAMP signaling in *SLC39A8*-deficient cells. These results suggest that *SLC39A8*-deficient cells cannot mount the proper response to external stimuli. The cAMP signaling in the brain has been shown to mediate various neural processes, including cell survival, neurogenesis, synaptic plasticity, learning, and memory (69–72). Given the observed decrease in neurogenesis and increase in apoptosis in the *Slc39a8*-NSKO cerebellum (Figure 3), exploring the potential link between cAMP-mediated signaling, neurogenesis, and apoptosis in future studies would be interesting. Furthermore, we observed significant downregulation of CREB-regulated genes (*Nr4a1*, *Nr4a2*, and *Nr4a3*), encoding nuclear receptors (73) with essential regulatory functions in the CNS (74–76). These encoded proteins act as transcription factors and play essential roles in various neural processes. Notably, NR4A2 has been extensively studied for its involvement in the development and maintenance of dopaminergic neurons (77) and its association with PD (78) and schizophrenia (79). Our data suggest a link between SLC39A8, Mn levels, and cAMP signaling pathways, underscoring therapeutic potential in Mn deficiency–induced neurodevelopmental conditions. Further studies are needed to examine how brain Mn contributes to cAMP signaling pathways involved in brain development and function.

Our findings align with recent research demonstrating that SLC39A8 plays a critical role in Mn uptake in the brain, particularly via the blood-brain barrier, using *Slc39a8*-inducible global knockout mice (80). Their radiotracer studies revealed that loss of SLC39A8 reduces Mn accumulation specifically in the brain, corroborating our observation of impaired brain Mn uptake in *Slc39a8*-NSKO mice. Although their study focused on systemic mechanisms of Mn regulation, our work highlights how SLC39A8 deficiency in neurons directly influences cerebellar development, motor function, and neurogenesis. Together, these studies underscore the essential role of SLC39A8 in maintaining brain Mn homeostasis and suggest distinct but complementary pathways through which SLC39A8 supports brain function.

Our findings revealed a marked reduction in Mn levels and notable neuropathological alterations predominantly in the cerebellum of *Slc39a8*-NSKO mice. This observation raises important questions about the unique susceptibility of the cerebellum to Mn dysregulation. The cerebellum’s high metabolic activity (81) and its critical role in motor coordination and learning (82) may make it particularly vulnerable to Mn dysregulation. Further, PCs, a dense population of neurons within the cerebellum, could be especially sensitive to Mn levels, which likely contributes to the dominance of the observed effects in this brain region. In conclusion, our study highlights the substantial impact of SLC39A8 on Mn uptake and cerebellar function. Future research should explore potential compensatory mechanisms in other brain regions and investigate therapeutic strategies to mitigate Mn deficiency in the cerebellum. Understanding these pathways could lead to better insights into the broader implications of Mn dysregulation in neurological disorders.

## Methods

### Sex as a biological variable.

Both female and male mice were studied.

### Animals.

For neuron-specific deletion of *Slc39a8*, *Slc39a8^fl/fl^* mice on a C57BL/6J background (European Mouse Mutant Archive, EM: 05285) were crossed with *Syn-Cre* mice on a C57BL/6J background (Jackson Laboratory) (Figure 1A). The *Slc39a8-flox* allele was detected by PCR using the following primers: forward: 5′-AAGGCGCATAACGATACCAC-3′ and reverse: 5′-CCGCCTACTGCGACTATAGAGA-3′. The *Syn-Cre* transgenic allele was detected using the following primers: forward: 5′-CTCAGCGCTGCCTCAGTCT-3′ and reverse: 5′-GCATCGACCGGTAATGCA-3′. Mice were group-housed in ventilated cage racks, maintained on a 12-hour light/12-hour dark cycle with controlled temperature and humidity, and provided the standard rodent diet of our institution (PicoLab Laboratory Rodent Diet 5L0D) and water ad libitum. Some of the metal content of this diet is as follows: 240 parts per million (ppm) iron, 76 ppm zinc, 70 ppm manganese, 13 ppm copper, and 0.41 ppm selenium. The manufacturer’s data sheet for this diet can be accessed from https://www.labdiet.com/getmedia/80040681-11e7-4e1b-a0ea-a4b5299fa8d3/5L0D.pdf?ext=.pdf All mice in the same experiment were from an age-matched, cohoused cohort.

### Metal measurements.

Tissue samples were analyzed for metals by ICP-MS, as previously described (83, 84). Briefly, tissue samples taken from mice were digested with 2 mL/g total wet-weight nitric acid for 24 hours and then digested with 1 mL/g total wet-weight hydrogen peroxide for 24 hours at room temperature. Specimens were preserved at 4°C until metals were quantified. Ultrapure water was used for the final sample dilution.

### ^54^Mn absorption studies.

To assess radiotracer Mn absorption, mice were dosed with 0.1 μCi ^54^Mn (PerkinElmer) per gram body weight via oral-intragastric gavage or tail vein injection. Mice were killed at 1 hour, after which ^54^Mn-associated radioactivity (counts per minute) in the brain regions and blood was determined using a gamma counter (Hidex), as previously described (38, 85).

### Immunohistochemistry.

Mouse brains were fixed in 10% neutral buffered formalin at 4°C overnight. H&E analysis was performed in paraffin-embedded brain sections (5 μm). Sections were processed for PC-specific immunohistochemical staining using an anti–calbindin D antibody (MilliporeSigma, C9848). Signals were visualized using a Vectastain avidin-biotin peroxidase complex kit (Vector Laboratories) and DAB (Vector Laboratories). Images were obtained from 3 midsagittal sections of wild-type (*n* = 3) and *Slc39a8*-NSKO (*n* = 3) mice. The dendritic length and the number of PCs were analyzed by using ImageJ (National Institutes of Health).

### BrdU labeling and TUNEL staining.

To examine the granule cell precursor proliferation in the cerebellum, the mice were injected with 100 mg/kg BrdU (BD Biosciences) 24 hours before being sacrificed. Midsagittal sections were immunostained with BrdU antibody (Abcam, ab6326) at 4°C overnight. The number of BrdU-positive cells in the EGL of the cerebellar cortex of wild-type (*n* = 3) and *Slc39a8*-NSKO (*n* = 3) mice was quantified in ImageJ. Cells undergoing apoptosis were identified by the TUNEL method as previously described (86). The TUNEL staining was performed using an in situ Cell Death Detection Kit, TMR red (Roche). After deparaffinization and rehydration, sections were permeabilized with 10 μg/mL Proteinase K solution (catalog P8107S, New England Biolabs) and incubated with the freshly prepared TUNEL reaction mixture in a dark and humidified chamber for 1 hour at 37°C. Nuclei were stained with DAPI (Thermo Fisher Scientific, D1306). TUNEL-positive cells were counted in 6 randomly selected fields per animal. Average values were taken from 3 animals of each genotype.

### Golgi-Cox staining and morphological analyses.

Brains from mice were dissected and incubated in a modified Golgi-Cox solution for 2 weeks at room temperature. The remaining procedure of Golgi-Cox immersion, cryosectioning, staining, and coverslipping was performed as described previously (87, 88). Three animals were used per genotype per sex, and cerebellum per animal was quantified. Quantification was done using commercially available software, NeuroLucida (v10, Microbrightfield), installed on a Dell PC workstation that controlled a Zeiss Axioplan microscope with a charge-coupled device camera (1,600 × 1,200 pixels) and with a motorized *x*, *y*, and *z* focus for high-resolution image acquisition (×100 oil immersion) and quantifications. The morphological analyses included dendritic lengths, spine counts, spine density, and numbers. All sample genotypes were masked to the analysts throughout the analysis.

### Behavioral assay.

All behavioral procedures were performed by investigators masked to the genotype of each group or the nature of the intervention. To decrease stress related to procedures, all animals were first habituated to handling by the experimenter and to the procedure room for at least 1 hour before testing. For hind limb clasping assessment, a mouse was gently removed from the cage and suspended by the tail for 5–10 seconds. The clasping score was scored as previously described (89). Two independent investigators scored all mice, and any mouse where the 2 scores differed by more than 1 point was rescored again. For rotarod assay, animals were placed on an accelerating rotarod (IITC Life Science) with the standard protocol with increasing speed from 4 to 40 rpm over 300 seconds, and the time taken by the animal to fall from the rod was recorded. The test was repeated twice and averaged to represent a score. For the balance beam test, the balance beam apparatus is a flat, 1-meter beam with a width of 5 mm that is suspended 50 cm above the table on 2 support poles. One side has a transparent square perch, while the other is opaque, containing a hut and nesting material. Mice were trained for 3 consecutive days at 5 weeks of age by sitting in the hut for 5 minutes and run across 3 different sections of the beam with a minute rest in between each run. Mice began runs during their sixth week, where they were placed in the hut for 5 minutes, then ran thrice with a minute rest between each run, with the maximum time to cross being 20 seconds. The 3 runs were averaged together. Weight was also recorded. For open field assay, mice were habituated for 1 hour in the test room. Each mouse was placed singly in the center of the open field apparatus. Video recording began 3 seconds after the mouse was detected in the arena and continued for 30 minutes. When the test was finished, mice were removed and returned to their home cage. The open field chambers were wiped down after each trial with 70% ethanol and paper towels to eliminate odor.

### RNA-Seq.

RNA-Seq was performed as previously described (83). Briefly, total RNA was isolated from the cerebellum per mouse using the RNeasy Mini Kit (QIAGEN), and 3 mice (*n* = 3 biological replicates) were used per genotype. RNA concentrations were measured with an Epoch Microplate Spectrophotometer (BioTek Instruments). RNA integrity was assessed with an Agilent Bioanalyzer 2100 using a Nano 6000 assay kit (Agilent Technologies). An RNA integrity number greater than 7.2 was considered the minimum requirement for library preparation. RNA was reverse-transcribed into cDNA using oligo-dT, and cDNA libraries were generated with an NEBNext Ultra II RNA Library Prep Kit (New England Biolabs E7775). An insert size of 250–300 bp was used for cDNA library preparation. Libraries were sequenced on the Illumina NovaSeq 6000 platform with a 150 bp paired-end mode. Reference genome and annotation files were downloaded from Ensembl, and RNA-Seq data were aligned to the reference genome using the Spliced Transcripts Alignment to a Reference (STAR) software (90). The DESeq2 package was used for differential expression analysis.

### qPCR.

Purified RNA was reverse-transcribed with SuperScript III First-Strand Synthesis System (Invitrogen, Thermo Fisher Scientific), as previously described (83). The qPCR was performed using the Power SYBR Green PCR Master Mix (Applied Biosystems). The mRNA was normalized using 36B4. The primers used for qPCR are listed in Supplemental Table 3 and were all purchased from Integrated Genomics Technologies.

### Cell culture.

All culture media and supplements were purchased from Thermo Fisher Scientific. Heat-inactivated fetal bovine serum was purchased from MilliporeSigma. SH-SY5Y cells were grown in DMEM containing 10% fetal bovine serum, penicillin (100 IU/mL), and streptomycin (100 mg/mL) at 37°C in a humidified, 5% CO_2_ incubator.

### Luciferase reporter assays.

Luciferase reporter assays were used to assess the role of SLC39A8 in the transcriptional response to the cAMP-mediated signaling pathway. The pGL3-Luciferase reporter vector containing 3 CRE sequences or mutated sequences was used as previously described (54). SH-SY5Y cells were transfected with 100 ng of the CRE-TK-Luciferase constructs, 1 ng of a CMV-Renilla construct, and 100 ng of either scramble (MilliporeSigma, SIC001–10NMOL) or *hSLC39A8* siRNA (MilliporeSigma, SASI_Hs02_00355573) using Lipofectamine 3000 (Invitrogen), as described previously (15). Two days later, cells were exposed to 30 μM forskolin or an equal volume of DMSO for 8 hours. Then, cells were harvested for Luciferase analysis using the Promega Dual Luciferase Assay System, and the assay was performed as previously described (54). The ratio between Luciferase and Renilla expression was normalized to the empty plasmid (TK-Luciferase only), and then Luciferase expression was reported relative to the DMSO, control siRNA condition.

### Statistics.

Statistical analysis was performed using GraphPad Prism 8 software. Data are presented as individual values and represent the mean ± SEM. To compare the 2 groups, 2-tailed *P* values were calculated using an unpaired *t* test. To compare more than 2 groups, *P* values were calculated using 1-way ANOVA with Tukey’s multiple comparisons test or 2-way ANOVA with Bonferroni’s multiple comparisons test. Outliers were identified using the GraphPad ROUT method (*Q* = 1%). Values of *P* < 0.05 were considered statistically significant. Asterisks in graphs, wherever present, denote statistically significant differences.

### Study approval.

All animal studies were performed in accordance with the guidelines in the *Guide for the Care and Use of Laboratory Animals* (National Academies Press, 2011) of the National Institutes of Health. The protocol (protocol number: PRO00010742) was approved by the University Committee on Use and Care of Animals at the University of Michigan.

### Data availability.

All nonsequencing data are contained within the Supporting Data Values XLS file. The RNA-Seq data are available via the NCBI Gene Expression Omnibus accession number GSE217704.

## Author contributions

YAS was responsible for conceptualization. EKC, LA, YP, ABC, APL, SI, and YAS were responsible for formal analysis. SI and YAS were responsible for funding acquisition. EKC, LA, YP, ABC, APL, SI, and YAS were responsible for conducting experiments. EKC, LA, YP, ABC, APL, SI, and YAS were responsible for analyzing data. YAS was responsible for project administration and supervision. YAS was responsible for writing the original draft. All authors were responsible for the review and editing of the manuscript.

## Supplementary Material

Supplemental data

Supporting data values

## Figures and Tables

**Table 1:**
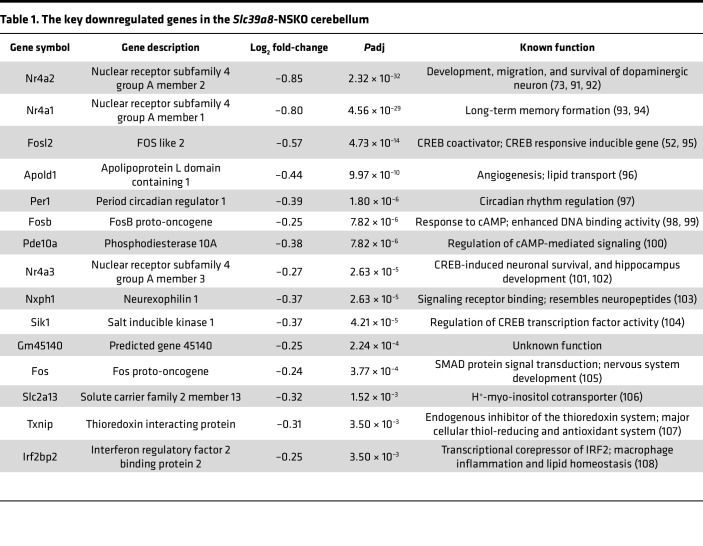
The key downregulated genes in the *Slc39a8*-NSKO cerebellum

## Version 1 10/22/2024

Electronic publication
