# Supplementary material for: Neuronal SLC39A8 deficiency impairs cerebellar development by altering manganese homeostasis
Source: JCI Insight. 2024 Oct 22;9(20):e168440. doi: 10.1172/jci.insight.168440 (PMC11530126; doi:10.1172/jci.insight.168440)
Supplement: Supplemental data [file jciinsight-9-168440-s026.pdf]

Supplemental information

Supplemental Figures

Figure S1

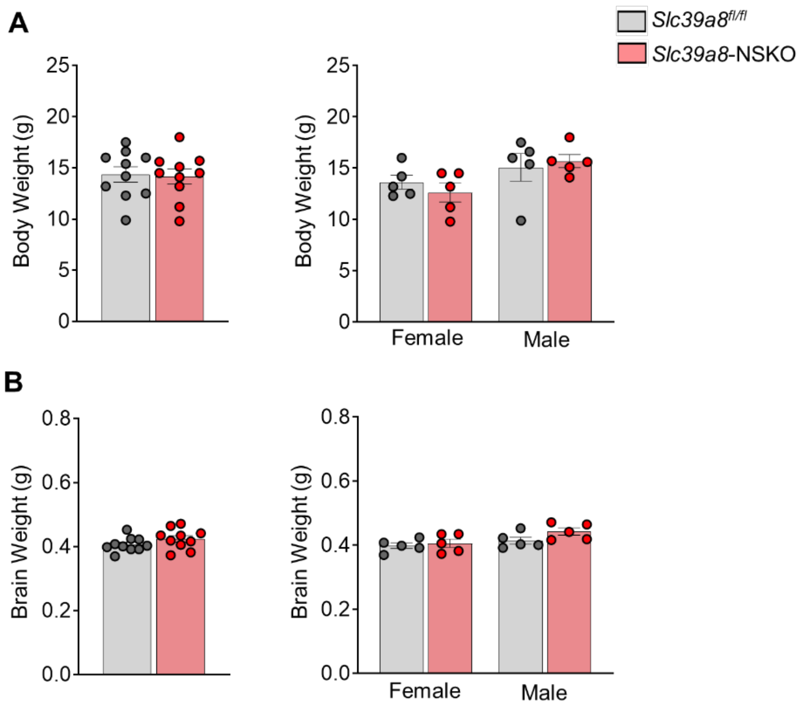

Figure S1. Physiological characteristics of *Slc39a8*-NSKO mice
(A) Mouse weight in 4-week-old male and female control and *Slc39a8*-NSKO mice.
(B) Brain weight in 4-week-old male and female control and *Slc39a8*-NSKO mice.
Data are presented as individual values and represent the mean ± SEM.

Figure S2

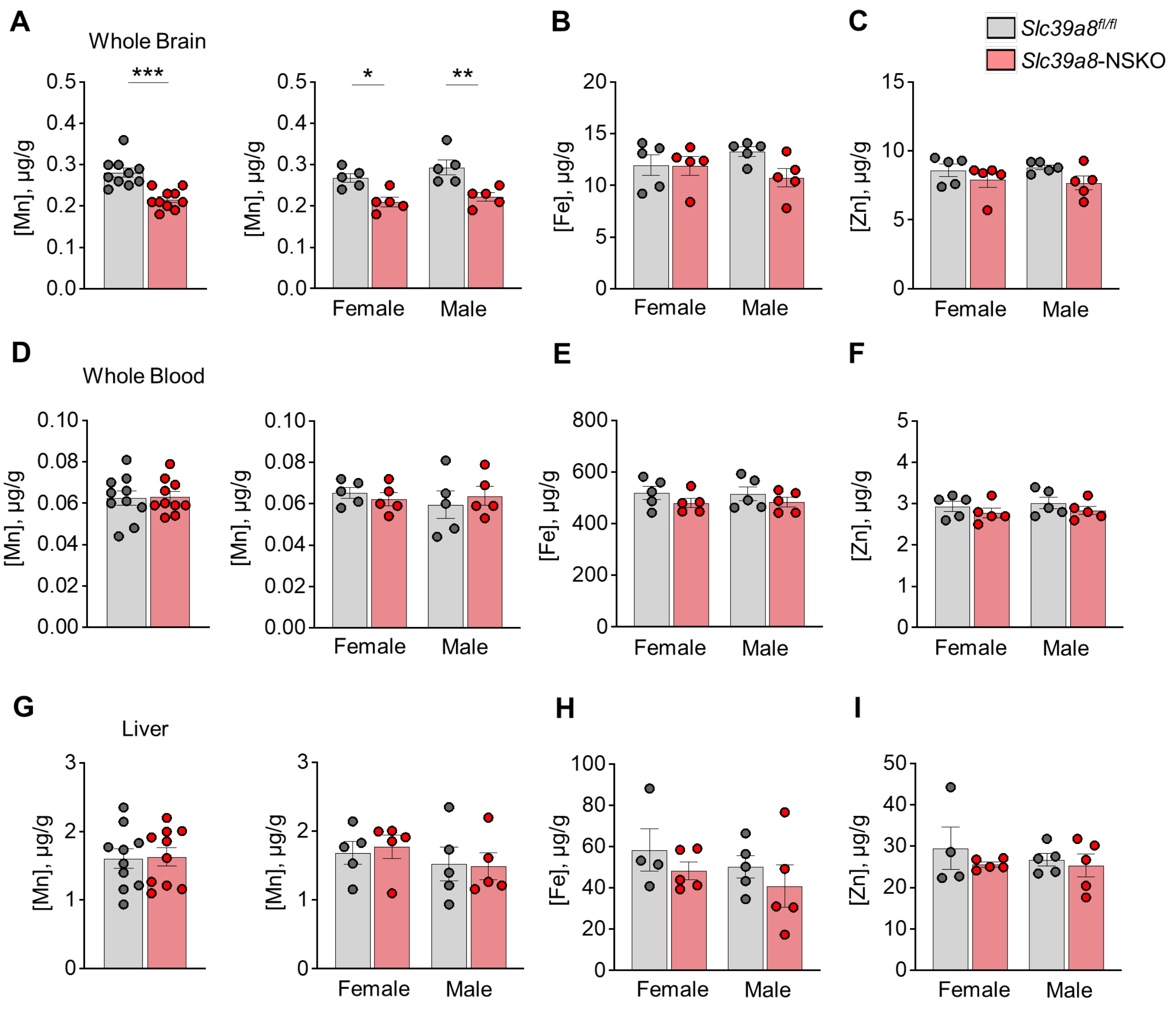

Figure S2. Neuronal *Slc39a8* deletion leads to brain Mn deficiency
(A−C) ICP-MS analysis of Mn (A), Fe (B), and Zn (C) levels in whole brain from 4-week-old male and female control and *Slc39a8*-NSKO mice.
(D−F) ICP-MS analysis of Mn (D), Fe (E), and Zn (F) levels in whole blood from 4-week-old male and female control and *Slc39a8*-NSKO mice.
(G−I) ICP-MS analysis of Mn (G), Fe (H), and Zn (I) levels in liver from 4-week-old male and female control and *Slc39a8*-NSKO mice.
Data are presented as individual values and represent the mean ± SEM. * $P < 0.05$, ** $P < 0.01$, and *** $P < 0.001$.

Figure S3

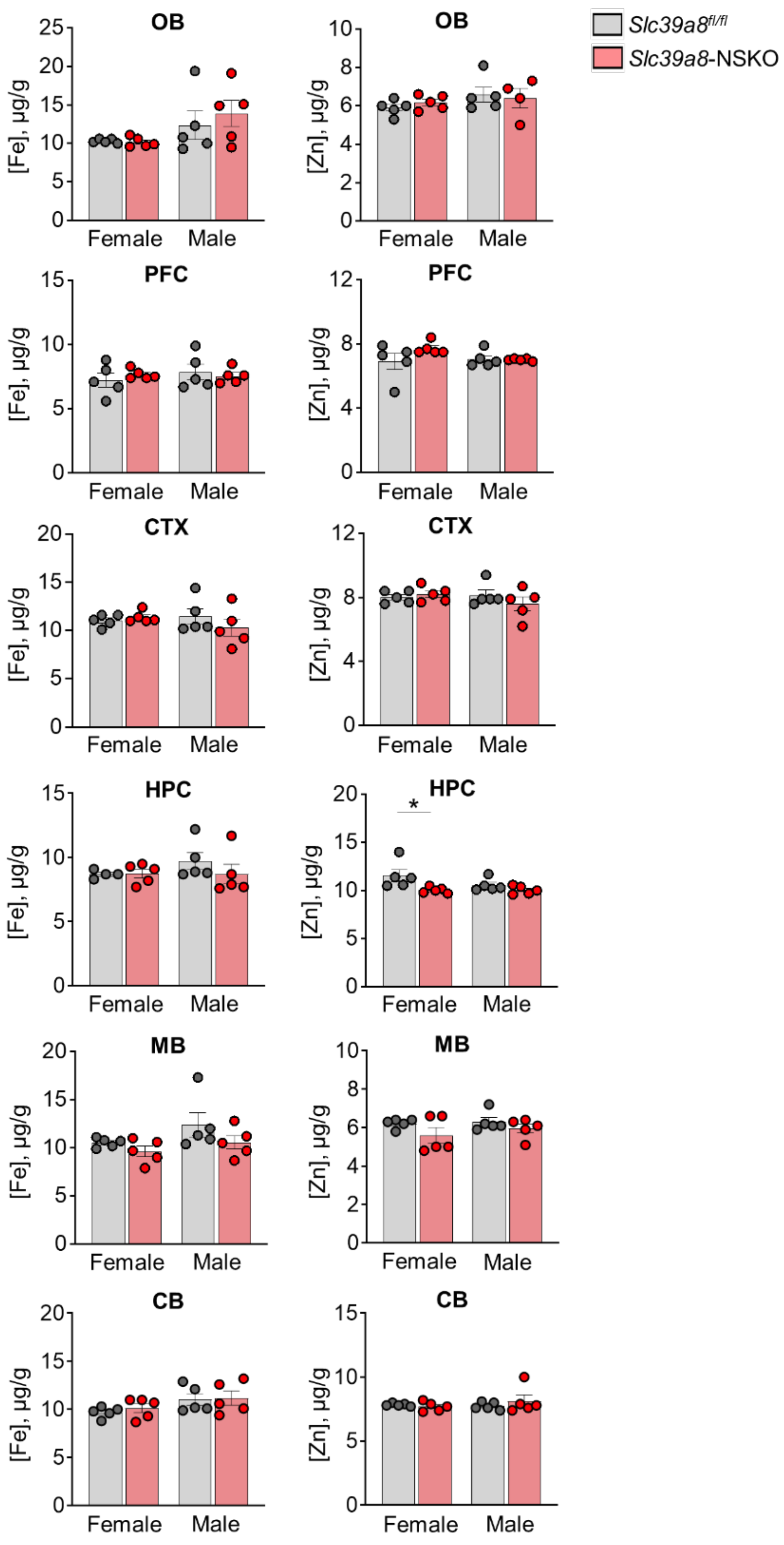

Figure S3. Loss of *Slc39a8* in neurons results in little impacts on iron and zinc levels in the brain region.
ICP-MS analysis of iron and zinc levels in olfactory bulbs (OB), prefronatal cortex (PFC), cortex (CTX), hippocampus (HPC), midbrain (MB), and cerebellum (CB) from 4-week-old male and female control and *Slc39a8*-NSKO mice.
Data are presented as individual values and represent the mean ± SEM. * $P < 0.05$.

Figure S4

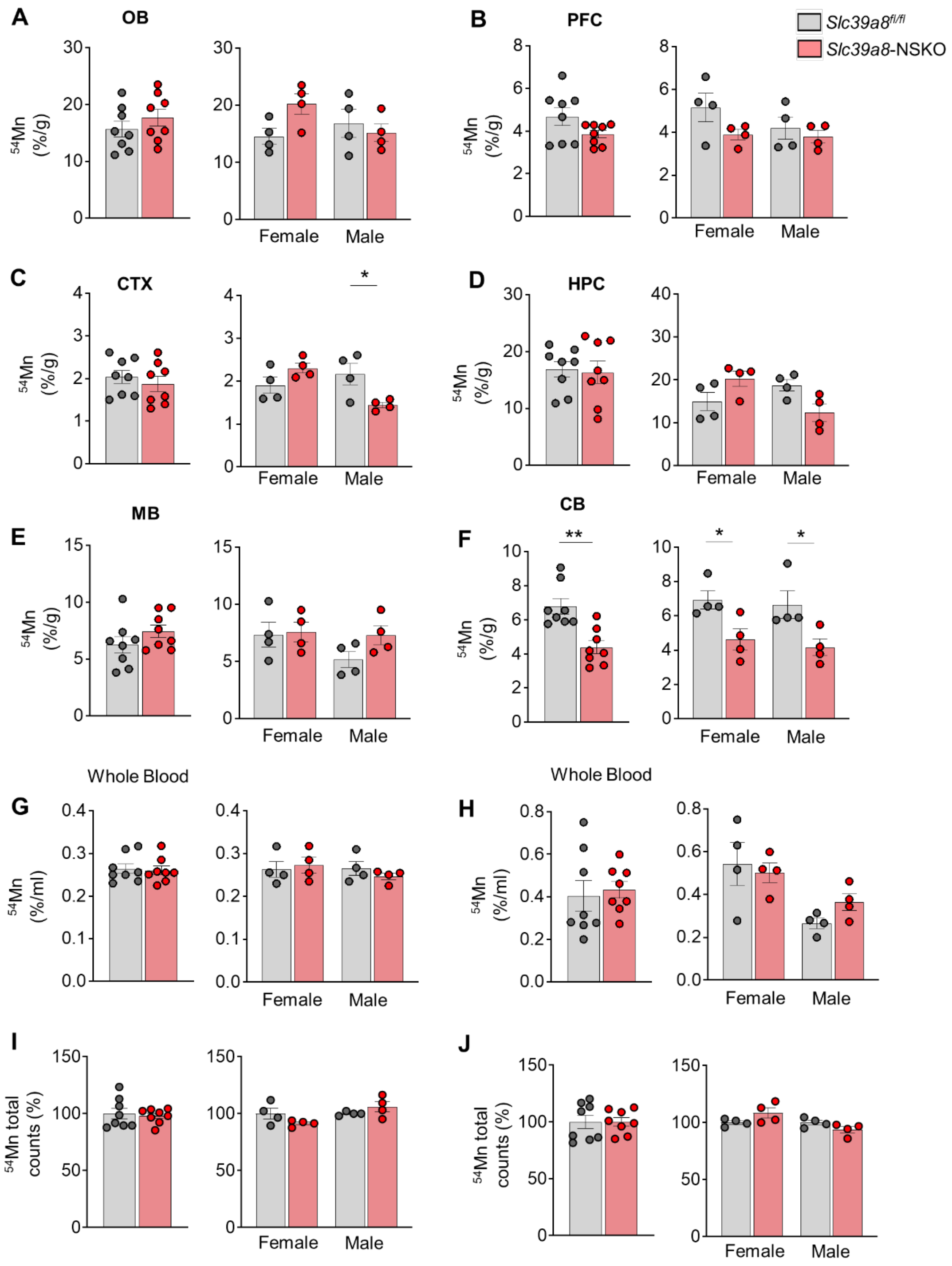

Figure S4. Neuronal *Slc39a8* deletion results in impaired brain Mn uptake.
(A-F) Control and *Slc39a8*-NSKO mice at 4 weeks of age were administered 0.1 µCi [^{54}Mn]$MnCl_2$ per gram body weight via oral-gastric gavage. Brain regions were collected at 1 hr, and cpm was determined by γ-counting. Levels of ^{54}Mn in olfactory bulbs (OB) (A), prefrontal cortex (PFC) (B), cortex (CTX) (C), hippocampus (HPC) (D), midbrain (MB) (E), and cerebellum (CB) (F) from 4-week-old male and female control and *Slc39a8*-NSKO mice.
(G, H) Blood was collected at 1 hr after intravenous injection (G) or after oral gavage (H), and blood counts per min (cpm) were determined by γ-counting.
(I, J) Levels of total ^{54}Mn between control and *Slc39a8*-NSKO mice for intravenous injection (I) or for oral gavage (J).
Data are presented as individual values and represent the mean ± SEM. * $P < 0.05$ and ** $P < 0.01$.

Figure S5.

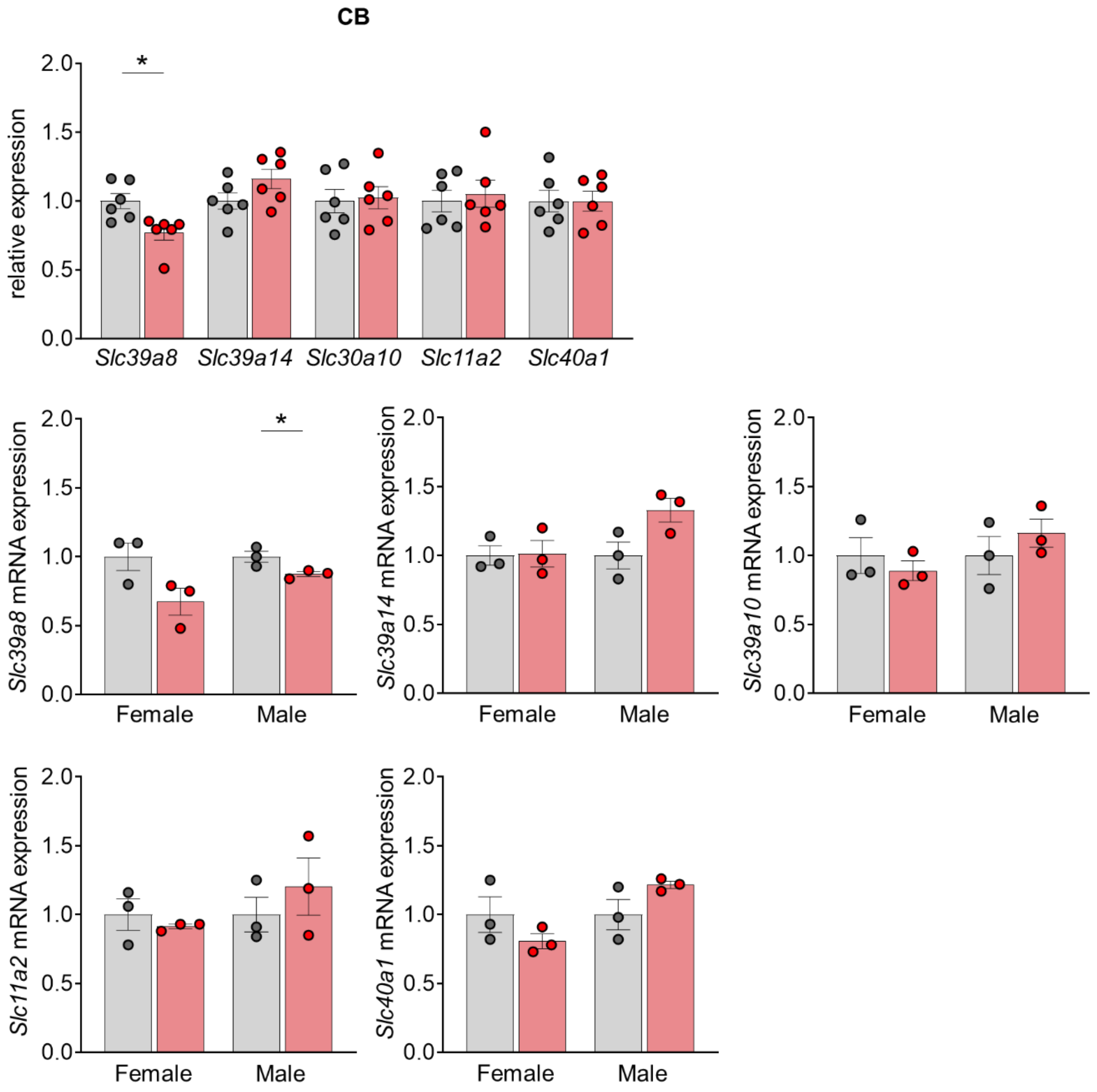

Figure S5. Transporter RNA levels were measured in cerebellum (CB) from 4-week-old male and female control and *Slc39a8*-NSKO mice.

Figure S6

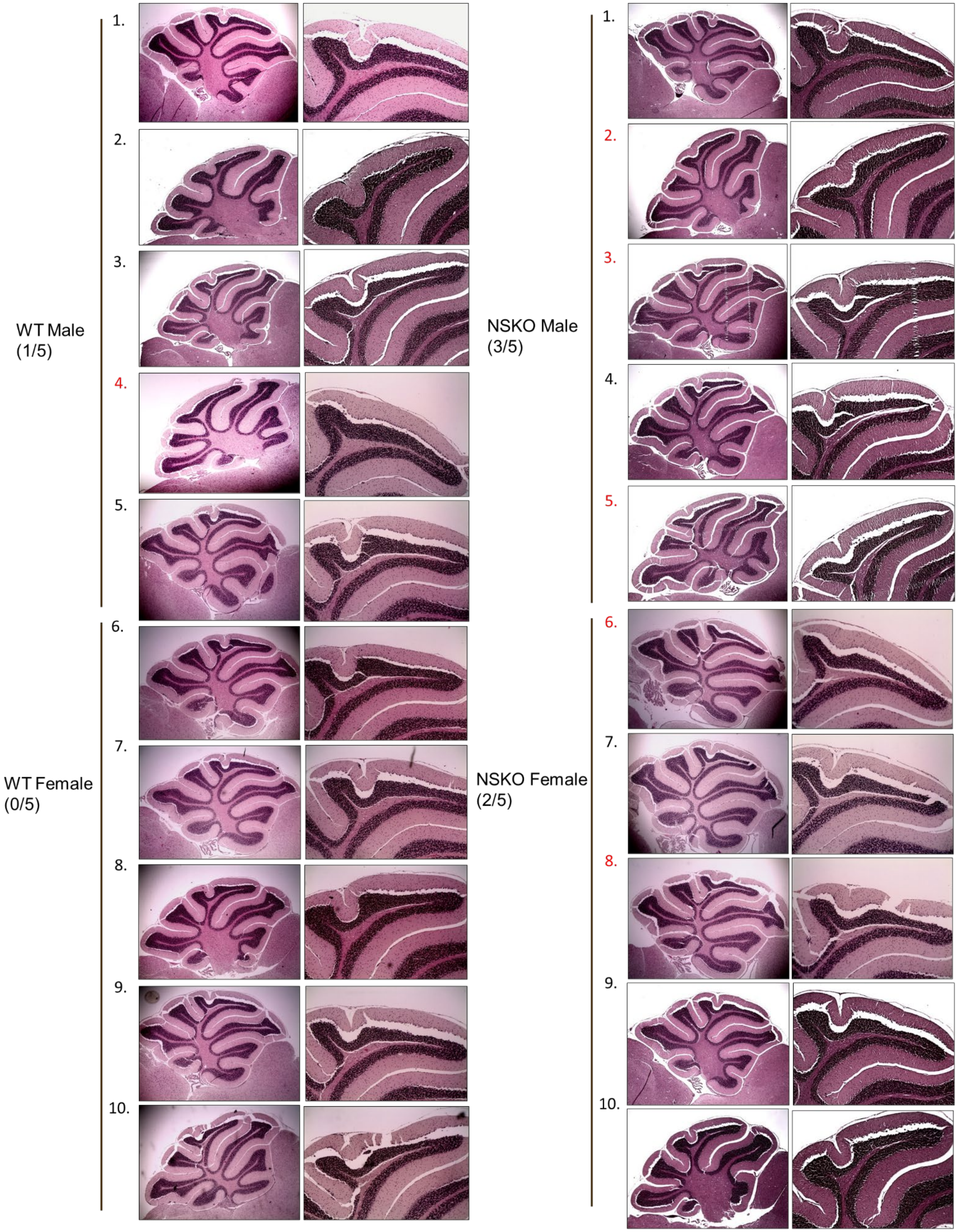

Figure S6. H&E-stained sagittal sections of paraffin-embedded mouse brains from 4-week-old control and *Slc39a8*-NSKO mice. Samples with morphological defects are highlighted red.

Figure S7

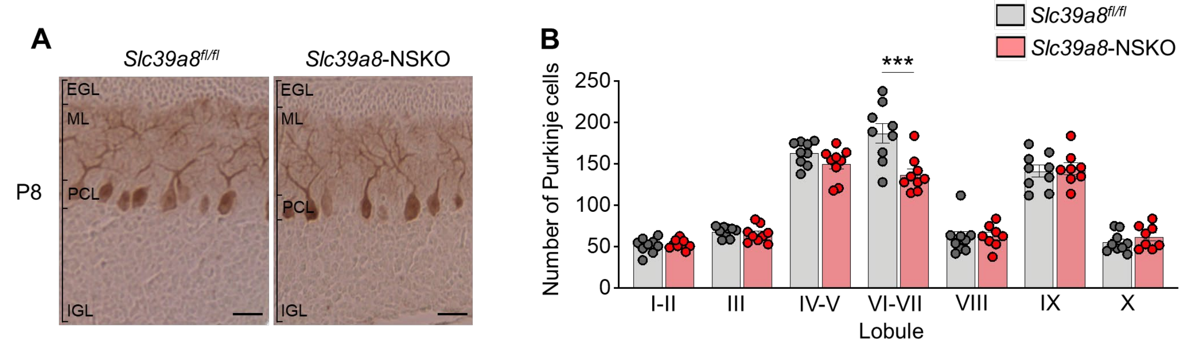

Figure S7. Morphological defects in *Slc39a8*-NSKO cerebellum.
(A) Immunohistochemical staining of cerebellar Purkinje cells (PCs) with anti-calbindin antibody. PC morphologies of *Slc39a8*-NSKO mice at P8. EGL, external granule cell layer; ML, molecular layer; PCL, Purkinje cell layer; IGL, internal granule cell layer. Scale bars: 25 μm
(B) Number of PCs at P8.
Data are presented as individual values and represent the mean ± SEM. *** $P < 0.001$.

Figure S8

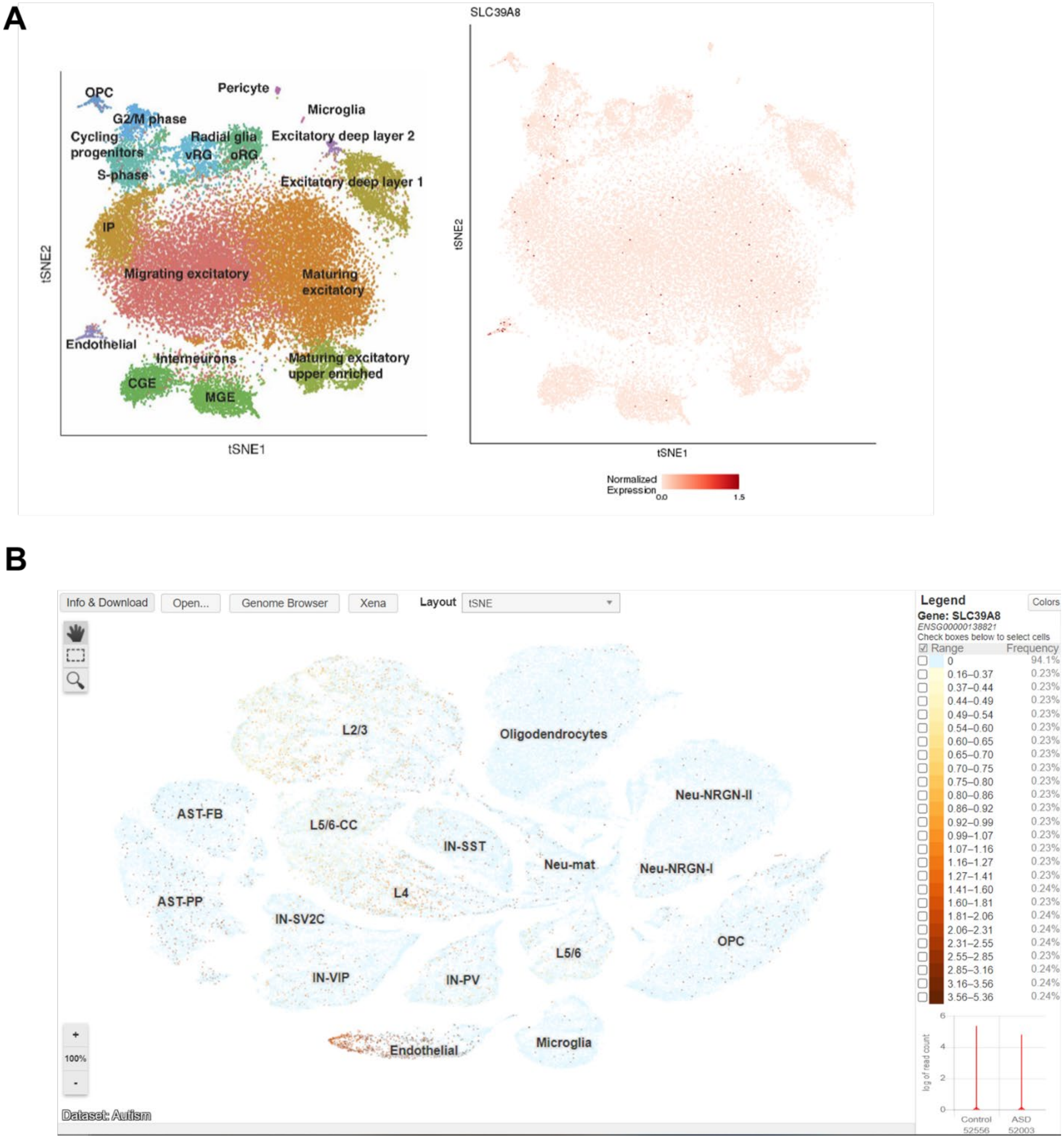

Figure S8. SLC39A8 mRNA expression from single-cell transcriptome analysis from human brain (1, 2).

Supplementary Table S1. The 36 downregulated and upregulated genes in the *Slc39a8*-NSKO cerebellum.

Gene symbol	Gene description	log2 fold-change	*P*adj
**Down-regulated**			
Nr4a2	Nuclear Receptor Subfamily 4 Group A Member 2	-0.85	2.3E-32
Nr4a1	Nuclear Receptor Subfamily 4 Group A Member 1	-0.80	4.6E-29
Fosl2	FOS Like 2	-0.57	4.7E-14
Apold1	Apolipoprotein L Domain Containing 1	-0.44	1.0E-09
Per1	Period Circadian Regulator 1	-0.39	1.8E-06
Fosb	FosB Proto-Oncogene	-0.25	7.8E-06
Pde10a	Phosphodiesterase 10A	-0.38	7.8E-06
Nr4a3	Nuclear Receptor Subfamily 4 Group A Member 3	-0.27	2.6E-05
Nxph1	Neurexophilin 1	-0.37	2.6E-05
Sik1	Salt Inducible Kinase 1	-0.37	4.2E-05
NA	Predicted gene 45140	-0.25	2.2E-04
Fos	Fos Proto-Oncogene	-0.24	3.8E-04
Slc2a13	Solute Carrier Family 2 Member 13	-0.32	1.5E-03
Txnip	Thioredoxin Interacting Protein	-0.31	3.5E-03
Irf2bp2	Interferon Regulatory Factor 2 Binding Protein 2	-0.25	3.5E-03
Ddit4	DNA Damage Inducible Transcript 4	-0.28	3.5E-03
Klf2	Kruppel Like Factor 2	-0.30	3.5E-03
Npas4	Neuronal PAS Domain Protein 4	-0.25	4.1E-03
Arrdc3	Arrestin Domain Containing 3	-0.26	5.1E-03
Nab1	NGFI-A Binding Protein 1	-0.29	5.2E-03
Glcci1	Glucocorticoid Induced 1	-0.30	7.3E-03
Rnf122	Ring Finger Protein 122	-0.29	7.4E-03
Kcnk10	Potassium Two Pore Domain Channel Subfamily K Member 10	-0.26	1.5E-02
Uncx	UNC Homeobox	-0.28	1.6E-02
Coq10b	Coenzyme Q10B	-0.27	1.8E-02
Creb5	CAMP Responsive Element Binding Protein 5	-0.28	2.3E-02
Ovca2	OVCA2 Serine Hydrolase Domain Containing	-0.27	3.1E-02
Gm42715	predicted gene 42715	-0.27	3.1E-02
Tob2	Transducer of ERBB2	-0.23	5.0E-02
**Up-regulated**			
Myh6	Myosin Heavy Chain 6	0.34	4.0E-11
ENSMUSG00000116417	NA	0.15	3.2E-04
Tnnt2	Troponin T2	0.17	9.7E-03
Zxda	zinc finger, X-linked, duplicated A	0.11	1.1E-02
Capn11	Calpain 11	0.14	2.3E-02
1700025G04Rik	RIKEN cDNA 1700025G04 gene	0.24	2.7E-02
Sulf1	Sulfatase 1	0.27	3.2E-02

Supplementary Table S2. Chow/Mn content variation in studies using *Slc39a8* A391T KI mice.

Study	Chow Manufacturer; Cat#	Mn content	Other metals
Sunuwar et al., 2021 (3)	Harlan Teklad Global 18% Protein Extruded Diet; #2018SX	100 pm	NA
Nakata et al., 2020 (4)	NA	NA	NA
Mealer et al., 2022 (5)	TestDiet; #5755	65 ppm	NA
Li et al., 2022 (6)	Regular sterilized rodent chow	NA	NA
Verouti et al., 2022 (7)	Provimi Kliba AG; #2223	12 mg/kg	Iron 65 mg/kg, zinc 45 mg/kg, copper 6 mg/kg, iodine 0.6 mg/kg, selenium 0.2 mg/kg

Supplemental Table S3. Primer sequences used in this study.

**Primer name**	**Sequence**
Nr4a2	TGAATGAAGAGAGCGGACAA
	TGTCGTAATTCAGCGAAGGA
Nr4a3	AAACTTGCAGAGCCTGAACC
	CTGGTGGTCCTTTAAGCTGC
Apold1	CCGTCCTGAAGGCCAAGATT
	AGAAAAACAACGCTGCGTCC
Per1	TGTCCGTCACCAGTCAGTGT
	CCAGGCAGGTCTTCCATC
Fosb	GTGAGAGATTTGCCAGGGTC
	AGAGAGAAGCCGTCAGGTTG
Myh6	ACGGTGACCATAAAGGAGGA
	TGTCCTCGATCTTGTCGAAC
36B4	TCATCCAGCAGGTGTTTGAC
	TACCCGATCTGCAGACACAC

Reference

1. Polioudakis D, de la Torre-Ubieta L, Langerman J, Elkins AG, Shi X, Stein JL, et al. A Single-Cell Transcriptomic Atlas of Human Neocortical Development during Mid-gestation. *Neuron.* 2019;103(5):785-801.e8.
2. Speir ML, Bhaduri A, Markov NS, Moreno P, Nowakowski TJ, Papatheodorou I, et al. UCSC Cell Browser: visualize your single-cell data. *Bioinformatics.* 2021;37(23):4578-80.
3. Sunuwar L, Frkatović A, Sharapov S, Wang Q, Neu HM, Wu X, et al. Pleiotropic ZIP8 A391T implicates abnormal manganese homeostasis in complex human disease. *JCI Insight.* 2020;5(20).
4. T N, EA C, M K, H L, MK S, J Y, et al. A missense variant in SLC39A8 confers risk for Crohn's disease by disrupting manganese homeostasis and intestinal barrier integrity. *Proceedings of the National Academy of Sciences of the United States of America.* 2020;117(46).
5. Mealer RG, Williams SE, Noel M, Yang B, D'Souza AK, Nakata T, et al. The schizophrenia-associated variant in SLC39A8 alters protein glycosylation in the mouse brain. *Mol Psychiatry.* 2022;27(3):1405-15.
6. Li S, Ma C, Li Y, Chen R, Liu Y, Wan LP, et al. The schizophrenia-associated missense variant rs13107325 regulates dendritic spine density. *Transl Psychiatry.* 2022;12(1):361.
7. Verouti SN, Pujol-Giménez J, Bermudez-Lekerika P, Scherler L, Bhardwaj R, Thomas A, et al. The Allelic Variant A391T of Metal Ion Transporter ZIP8 (SLC39A8) Leads to Hypotension and Enhanced Insulin Resistance. *Front Physiol.* 2022;13:912277.
